# Dinoflagellates, a Unique Lineage for Retrogene Research

**DOI:** 10.3389/fmicb.2018.01556

**Published:** 2018-07-11

**Authors:** Bo Song, Sijie Chen, Wenbin Chen

**Affiliations:** ^1^Guangdong Provincial Key Laboratory for Plant Epigenetics, College of Life Sciences and Oceanography, Shenzhen University, Shenzhen, China; ^2^Key Laboratory of Optoelectronic Devices and Systems of Ministry of Education and Guangdong Province, College of Optoelectronic Engineering, Shenzhen University, Shenzhen, China; ^3^BGI-Shenzhen, Shenzhen, China; ^4^China National GeneBank, BGI-Shenzhen, Shenzhen, China

**Keywords:** retrogene, retroposition, dinoflagellate, spliced leader, genome evolution

## Abstract

The birth and evolution of retrogenes have played crucial roles in genome evolution. Dinoflagellates represent a unique lineage for retrogene research because the retrogenes can be reliably identified by the presence of a 22 nucleotide splice leader called DinoSL, which is post-transcriptionally added to the 5′ terminus of all mRNAs. Compared to studies of retrogenes conducted in other model genomes, dinoflagellate retrogenes can potentially be more comprehensively characterized because intron-containing retrogenes have already been detected. Unfortunately, dinoflagellate retrogene research has long been neglected. Here, we review the work on dinoflagellate retrogenes and show their distinct character. Like the dinoflagellate genome itself, dinoflagellate retrogenes are also characterized by many unusual features, including a high survival rate and large numbers in the genome. These data are critical complements to what we know about retrogenes, and will further frame our understanding of retroposition and its roles in genome evolution, as well as providing new insights into retrogene studies in other genomes.

## Introduction

Gene duplication is an essential source of novel genes along the evolutionary trajectory of organisms and can be mediated by either DNA or RNA intermediates. DNA-based duplications have been comprehensively studied in plants and animals in last decade because of increases in the amount of genomic data available. DNA duplication allows sequence variations to accumulate in one of the copies which can thus result in dramatic changes of the genotypes and phenotypes (Van de Peer et al., 2017). RNA-based duplication, which involves reverse transcription of an RNA intermediate and DNA integration, is different because the cDNA intermediate lacks the regulatory elements required for transcription. Consequently, a majority of the duplicated genes are “dead upon arrival,” and are termed retrocopies; surviving duplicates are termed retrogenes, and the process of inserting both is referred to as retroposition (Kaessmann et al., 2009). Compared to large-scale duplications of chromosomal sections, RNA-based duplication is small scale, a single gene at a time, and the limited survival rate of retrocopies results in a lower overall change to the genome and the phenotype. However, retrogenes that acquire regulatory elements can potentially allow organisms to adapt more quickly to environmental changes that alter the expression of specific genes, since the frequency of retroposition will increase as a function of transcript levels. Retrogene research has primarily focused on model organisms such as humans and fruit flies (Kaessmann et al., 2009), but some effort has also been made with other lineages including land plants, green algae (Jąkalski et al., 2016) and dinoflagellates (Slamovits and Keeling, 2008; Jaeckisch et al., 2011; Song et al., 2017).

Dinoflagellate chromatin has many distinct features, including permanently condensed chromosomes, liquid crystal DNA, a lack of nucleosomes and undetectable histones (Lin, 2011). These features led to the idea of the dinokaryon, a structure intermediate between eukaryotic and prokaryotic chromatin (Rizzo and Cox, 1977), although it is now clear dinoflagellates are firmly in the eukaryotic lineage. Recent genome sequencing has revealed another surprising feature – a large number of survived retrogenes whose origins coincide with times of important changes in genome evolution (Song et al., 2017). Retrogene research in dinoflagellates is greatly facilitated by an unusual trans-splicing mechanism which adds a 22 nucleotide DinoSL (Dinoflagellate Spliced Leader to the 5′ end of all mRNA in all dinoflagellates) (Zhang et al., 2007). This post-transcriptionally added DinoSL can serve as a tag enabling easy and reliable identification of retrogenes in dinoflagellate genomes.

Although retrogene research in various organisms had been previously reviewed (Kaessmann et al., 2009; Casola and Betrán, 2017) dinoflagellates were not included. In part, this is due to the fact that dinoflagellate genomes have only recently become available. However, retrogenes in dinoflagellates have been variously called recycling genes (Slamovits and Keeling, 2008), or SL-containing genes (Lin et al., 2015), making them difficult to find in literature searches for retrogenes. In this mini review, we present the current research on dinoflagellate retrogenes, compare the differences between retrogenes in dinoflagellate and other organisms, and discuss the advantages of using dinoflagellates as a model for retrogene research.

## Identification of Retrogenes

In other genomes, the identification of retrogenes is based on the lack of introns compared to their parental copies (reviewed in Casola and Betrán, 2017). Clearly, this identification method will fail for parental genes lacking introns. Furthermore, this strategy assumes retroposition after the splicing of introns, which may not always be the case. As a result, retrogenes with introns, either novel or inherited from their parents, have been excluded from previous analyses, leading to an underestimation of the numbers of retrogenes in most of the studied genomes.

In dinoflagellates, the addition of the 22-nt DinoSL [DCCGUAGCCAUUUUGGCUCAAG (D = U, A, and G)] to the 5′ termini of an mRNA (Zhang et al., 2007) provides a tag allowing identification of any gene that has been retroposed from mRNA. The identification of retrogenes in dinoflagellate genomes involves searching for DinoSL or its relicts upstream of protein-coding genes and is thus independent of the presence or absence of introns. Not only does this approach enable the detection of retrogenes with introns, but the number of DinoSL sequences allows determination of the number of times that retroposition has occurred. The presence of multiple DinoSL tags actually allowed retrogenes to also be identified from transcriptomic data (Slamovits and Keeling, 2008; Jaeckisch et al., 2011; Lee et al., 2014) even before dinoflagellate genomic data became available (Shoguchi et al., 2013; Lin et al., 2015; Aranda et al., 2016).

Slamovits and Keeling (2008) reported the discovery of DinoSL relicts, downstream from the usual dinoSL, in more than 100 cDNAs, ESTs or ORFs from 15 dinoflagellates ranging from *Oxyrrhis marina* to *Alexandrium tamarense*. Multiple DinoSL sequences were found in about 20% of the full length transcripts analyzed, and were taken as evidence of multiple retroposition events (Slamovits and Keeling, 2008). Interestingly, retrogenes were also detected in *Perkinsus marinus*, an early branching dinoflagellate lineage, suggesting retroposition has occurred widely during the evolution of dinoflagellates. An analysis of 238 *Alexandrium* (*A. tamarense*, *A. ostenfeldii*, *A. minutum*, and *A. catenella*) transcripts whose 5′ termini had been completely recovered as shown by the presence of full DinoSLs at their 5′ ends, revealed that 61 (25.6%) had multiple DinoSLs indicative of retroposition, and 17 (7.1%) had actually been retroposed more than once (Jaeckisch et al., 2011).

Genomic research in the dinoflagellates began with the release of several *Symbiodinium* genome sequences (Shoguchi et al., 2013; Lin et al., 2015; Aranda et al., 2016). *Symbiodinium* is a coral-symbiotic lineage with smallest known genome among the dinoflagellates. A large number of retrogenes (9,339 in *S. minutum* and 8,564 in *S. kawagutii*) were identified in these genomes (Song et al., 2017). This large number of retrogenes (22.3 and 23.2% of the total genes in *S. minutum* and *S. kawagutii* genomes, respectively) agrees remarkably well with the estimates derived from transcriptomic studies in *Alexandrium* (25.6%) (Jaeckisch et al., 2011) and in a pool of dinoflagellate ESTs (25%) (Slamovits and Keeling, 2008). The retrogenes recovered from the genomes greatly enlarged the reservoir of retrogenes and, more importantly, provided an opportunity for genome-wide and detailed analysis of retrogene character and function.

## Birth of Retrogenes

Compared to the retrogenes in other genomes, dinoflagellate retrogenes are substantially more abundant and, because of the presence of the retroposition DinoSL “tag,” can be readily distinguished from their parental copies thus allowing evolutionary analyses. The parental copies of retrogenes have similar sequences, have retained their introns, and have fewer DinoSLs than the retrogenes (Song et al., 2017). As with observations in other genomes, a majority of the dinoflagellate retrogenes are “orphans.” Only 6.4% of the retrogenes in *S. minutum*, and 9.8% in *S. kawagutii*, were successfully paired with their parental copies (Song et al., 2017), very close to the 8.5% value found in green algae (Jąkalski et al., 2016). Despite the large number of “orphan” retrogenes, there were still enough retrogenes with detectable parents (599 in *S. minutum* and 843 in *S. kawagutii*) to enable further analysis into the birth and evolution of retrogenes as well as into their functions.

Given the large number of retrogenes in dinoflagellate genomes, one obvious question is whether retroposition occurred continuously or was episodic throughout evolution. Previous evidence of the high activity of transposons implies retroposition is ongoing in *O. marina* (Lee et al., 2014). In contrast, the synonymous mutation rate of retrogenes suggested two separated episodes of extensive retroposition during the evolution of *Symbiodinium*. The first episode occurred about 60 million years ago (MYA), a time close to the Cretaceous-Paleogene boundary, when a number of catastrophic events occurred and led to dramatic climate change and global warming (Petersen et al., 2016). Coincidently, during this period, whole genome duplication (WGD) events frequently occurred in land plants (Van de Peer et al., 2017). The second episode occurred about 6 MYA, coinciding with the radiation of *Symbiodinium* species (LaJeunesse, 2005; Thornhill et al., 2014) and with another wave of WGDs in land plants that occurred in the late Micocene period (5.5–11.6 MYA). This latter was marked by an expansion of the C4 grasslands on land (Estep et al., 2014) and extinction of coralline read algae species in the ocean (Aguirre et al., 2000). It is intriguing that both episodes correspond to times of dramatic climate changes on earth (Petersen et al., 2016), and suggests climate change resulting in massive genome-shaping events may have facilitated adaptation to environmental changes through genome expansion.

## Implications of Retroposition

The coincidence between the two episodes of retroposition and crucial periods of *Symbiodinium* evolution suggests another question – what genes were retroposed and is their duplication likely to have promoted the evolution of *Symbiodinium*? Analyses of *S. minutum* and *S. kawagutii* genomes suggest that genes retroposed in the first episode are enriched in functions related to ion and protein transmembrane transport, while retrogenes in the second episode are enriched in groups related to photosynthesis and the establishment of symbiosis (Song et al., 2017). These retrogenes are present in large numbers, suggesting their parent genes were highly expressed at the time when retroposition occurred since highly expressed genes have a greater chance to be retroposed (Pavlicek et al., 2006). Therefore, genes related to transmembrane transport were likely expressed at a very high level during the first episode. Indeed, transmembrane transporters are generally enriched in the dinoflagellates (Aranda et al., 2016) suggesting gene duplication mediated by retroposition may have been important in the evolution of different species. The enrichment of genes related to photosynthesis in the second episode may reflect a response to the decreased level of CO_2_ (Estep et al., 2014). The low concentration of CO_2_ at this time was accompanied by substantial oscillations in temperature (Zhang et al., 2013) as well as regressions and transgressions of sea-level (Haq et al., 1987) which may have led to symbiont replacement in reef invertebrates (LaJeunesse, 2005). This replacement might have been facilitated by the activation and retroposition of genes involved in the establishment of symbiosis, which could thus have allowed *Symbiodinium* access to an expanded range of hosts.

**FIGURE 1:**
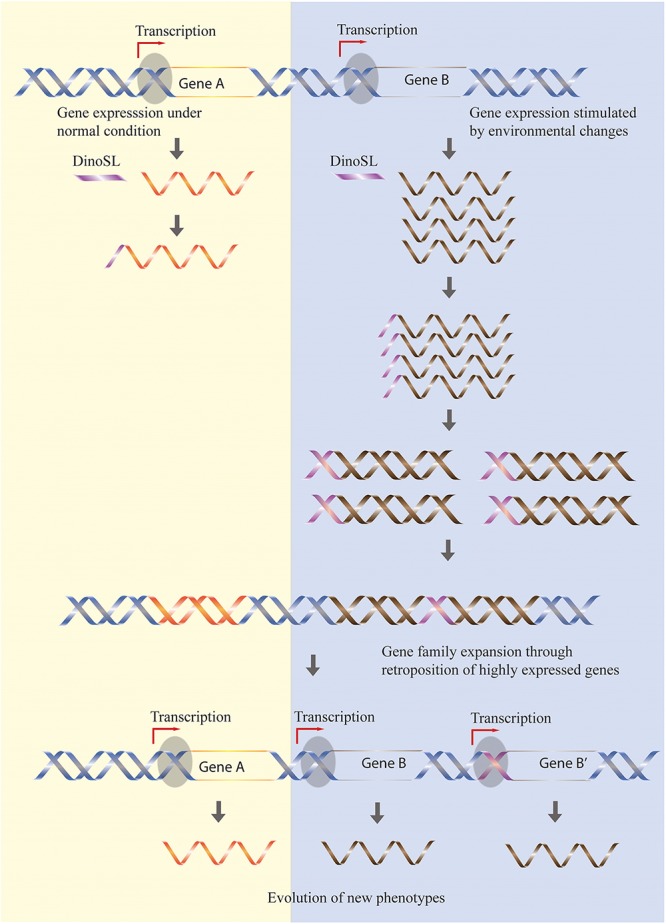
Retroposition mediates the expansion of gene families responsive to stress. Genes highly expressed by certain environmental conditions (gene B) have higher chances to be retroposed, so the increased expression level is fixed in the genome through an increased retrogene copy number. In contrast, gene families not stimulated by environmental stress (gene A) remain constant during evolution. The different rate of retroposition of these two classes of genes will thus allow accumulation of genes responsive to environmental changes.

The preference for the retroposition of highly expressed genes has the effect of “fixing” high expression levels of active genes, with an increase in gene copies constituting a self-re-enforcing model of dinoflagellate genome evolution (Song et al., 2017). Additional evidence supporting this model is the accumulation of genes related to stress responses in *Symbiodinium* genomes (Lin et al., 2015) since expression of these genes should have been stimulated by the dramatic changes in climate occurring when large-scale retroposition episodes were triggered during the evolutionary history of *Symbiodinium* (**Figure 1**).

## Mechanism of Retroposition

The high expression level of genes engaged in the process of “RNA-dependent DNA replication” during both the episodes of retroposition in *Symbiodinium* (Song et al., 2017) suggests that retroposition in dinoflagellates might have been mediated by retrotransposons. However, it is unknown which type has mediated the reverse transcription and retroposition. Both non-LTR and LTR retro-elements have been proposed to mediate retroposition in other organisms (Casola and Betrán, 2017). In dinoflagellates, retroposition is more likely mediated by the latter because the LTR-retrotransposon Ty1/copia is highly expressed in dinoflagellate cells (Lee et al., 2014) and its expression has been found to be activated by increased temperature (Chen et al., 2017). A possible scenario is that catastrophic events or changes in climate stimulated the activities of Ty1/copia retroelements which in turn triggered the large-scale retroposition events in dinoflagellates. Retroposition is rapid, as the retention of introns in retrogenes (Slamovits and Keeling, 2008; Song et al., 2017) suggests that genes have retroposed before the mRNAs are exported to the cytoplasm. Furthermore, retrogenes tend to be inserted into a locus near their parents (Song et al., 2017), suggesting retroposition can occur immediately after transcription.

One important difference between the retrogenes of dinoflagellates and other organisms is their high survival rate. A possible explanation for this difference is that the DinoSL added to the transcripts contains a potential promoter motif, TTT(G), which is then retroposed into genome together with coding sequences (**Figure 1**). This hypothesis is supported by several observations, first being that the DinoSL relicts located between -50 and -100 from the start codon [the usual length of 5′ UTR in dinoflagellates (Zhang et al., 2007; Kim et al., 2010)] are more conserved regardless of their ages. In addition, the potential promoter motif, TTT(G), is more conserved than other motifs in DinoSL relicts upstream of the retrogene. Lastly, there are few retrogenes containing multiple DinoSL sequences. This would be expected if the retroposed DinoSL served as a promoter, as the retrogene transcript would then contain only the post-transcriptionally added DinoSL. In contrast, an upstream promoter would result in many retrogene transcripts with tandem DinoSLs. A counterargument is that analyses of dinoflagellate ESTs show that younger (more 5′) DinoSL relicts had accumulated fewer mutations compared to the old ones (Slamovits and Keeling, 2008; Jaeckisch et al., 2011), although this may be due simply to the fact that any DinoSL relicts further upstream that were able to function as promoters would not be found in EST sequences.

## Concluding Remarks

Retrogenes have been extensively studied in model organisms including humans, mice, fruit flies, and rice. However, the results based on these studies might have underestimated the extent of retroposition because of the assumption that only mature (spliced) mRNAs would be retroposed. In the dinoflagellates, even retrogenes with introns can be identified in the genomes using the post-transcriptionally added DinoSL sequence as a hallmark of retroposition. The possibility that other organisms have ‘invisible’ retrogenes with introns is difficult to evaluate experimentally, however. One possible approach might involve machine learning, in which the dinoflagellate retrogenes are used to train a model for retrogene identification in other organisms. This will undoubtedly be facilitated by the sequencing of dinoflagellate genomes in other lineages. Transcriptomes indicate retrogenes are widespread across dinoflagellates, so it is reasonable to expect that other genomes will also have a large number of retrogenes.

Compared to model organisms, genomic resources are still rather poor for dinoflagellates, which greatly limits our understanding of the evolution and function of retrogenes. With the advancements in sequencing technology, assembly and annotation, it is anticipated that more and bigger dinoflagellate genomes will be released in near future. These will further refine the roles that retroposition played in genome evolution.

## Author Contributions

BS and WC conceived the work. BS, WC, and SC wrote the manuscript. BS and SC drew the figure.

## Conflict of Interest Statement

The authors declare that the research was conducted in the absence of any commercial or financial relationships that could be construed as a potential conflict of interest.
